# Effects of alcohol on gut microbiome in adolescent and adult MMTV-Wnt1 mice

**DOI:** 10.3389/fonc.2025.1557040

**Published:** 2025-07-16

**Authors:** Hui Li, Leeann Aguilar Meza, Shailesh K. Shahi, Zuohui Zhang, Wen Wen, Di Hu, Hong Lin, Ashutosh K. Mangalam, Jia Luo

**Affiliations:** ^1^ Department of Pathology, University of Iowa Carver College of Medicine, Iowa City, IA, United States; ^2^ Holden Comprehensive Cancer Center, University of Iowa, Iowa City, IA, United States

**Keywords:** alcohol misuse, breast cancer, gut dysbiosis, tumor promotion, Wnt1

## Abstract

**Introduction:**

Breast cancer is the most frequently diagnosed cancer in women worldwide. Alcohol consumption is a significant and modifiable risk factor, yet the mechanisms linking alcohol to breast cancer progression remain unclear. Recent evidence suggests that the gut microbiome—a complex ecosystem that modulates metabolism, immunity, and inflammation—may act as a mediator of alcohol-induced tumor promotion. We hypothesized that binge-like alcohol exposure induces gut dysbiosis, which in turn drives systemic inflammation and carcinogenic processes.

**Methods:**

We utilized MMTV-Wnt1 transgenic mice, a well-established model for mammary tumor development, along with wild-type FVB mice. Adolescent and adult female mice were administered binge-like doses of ethanol via intraperitoneal injection. Fecal samples were collected and analyzed by 16S rRNA gene sequencing to assess microbial diversity, composition, and taxonomic changes in response to alcohol exposure.

**Results:**

Binge-like alcohol exposure significantly reduced gut microbial richness in adult Wnt1 and FVB mice. In both adolescent and adult mice, alcohol markedly altered the composition of the gut microbiota across both strains. Differential abundance analysis identified specific microbial taxa significantly impacted by ethanol treatment, suggesting targeted perturbations of the gut microbial community.

**Conclusion:**

Our findings demonstrate that intraperitoneal binge-like alcohol exposure induces gut dysbiosis in both tumor-prone and wild-type mice. These alterations in the gut microbiome may contribute to the pro-inflammatory and tumor-promoting effects of alcohol in breast tissue. This study provides insights into the potential role of gut dysbiosis in alcohol-induced mammary tumor promotion and offers avenues for future research.

## Introduction

Alcohol consumption has emerged as a critical risk factor for breast cancer as many epidemiological and experimental studies have demonstrated a positive correlation between alcohol consumption and increased breast cancer risk (1–6). However, the cellular and molecular mechanisms underlying alcohol’s tumor promotion remain unclear. There are several proposed mechanisms. For example, alcohol consumption can elevate the estrogen levels in both premenopausal and postmenopausal women, which may contribute to the effect of alcohol on increased breast cancer risk (7–10). Alcohol and its metabolite, acetaldehyde, are both known to damage DNA and induce gene mutations (11, 12). Alcohol exposure can also promote the accumulation of excessive reactive oxygen species (ROS) and oxidative stress, which may promote mammary carcinogenesis and aggressiveness (5, 11). Recently, studies have shown that alcohol consumption may also affect the gut microbiome, an essential regulator of systemic inflammation, estrogen metabolism, and immune responses, suggesting a *novel* pathway through which alcohol may impact breast cancer risk and progression (13–15).

The gut microbiome is the complex community of microbes such as bacteria, viruses and fungi that reside in the gastrointestinal system and modulate the functions of local and distant organs through metabolic, immunologic and hormonal pathways (16–18). For instance, short-chain fatty acids produced by microbial fermentation of dietary fiber regulate immune responses and gut barrier function (19–21). Certain gut bacteria can influence the production of neurotransmitters such as serotonin and dopamine, thereby impacting brain function and behavior (22, 23). A disruption in the composition of the gut microbiome, known as gut dysbiosis, is characterized by reduced microbial diversity, loss of beneficial bacteria or overgrowth of harmful bacteria. Gut dysbiosis has been linked to a wide range of diseases including breast cancer (24, 25). Recent studies have suggested that gut dysbiosis plays a role in various aspects of breast cancer, including tumorigenesis, disease progression, metastasis and treatment outcome (26–29). For example, a gut microbiome profiling study conducted in the Midwestern United States revealed gut dysbiosis in breast cancer patients, characterized by the depletion of short-chain fatty acid-producing gut bacteria (30). Additionally, a pilot study reported associations between gut microbiome composition and breast tumor characteristics, such as receptor status, stage, and grade, as well as established breast cancer risk factors (31).

Emerging evidence has demonstrated that alcohol consumption can disrupt the gut microbiome and alcohol-induced gut dysbiosis is considered an early factor in alcohol-related disorders such as alcohol use disorders (AUD) and alcoholic-liver disease (ALD) (13–15, 32). The role of gut dysbiosis in alcohol-related breast cancer, however, has not yet been studied. Using a mammary tumorigenesis model of MMTV-Wnt1 transgenic mice, we have previously shown that alcohol exposure enhanced tumorigenesis and aggressiveness, with adolescent mice showing greater sensitivity to the effects of alcohol than adults (33). In this study, we aimed to determine whether alcohol exposure alters the gut microbiome in MMTV-Wnt1 mice prior to the onset of mammary tumorigenesis by analyzing changes in gut bacterial composition following alcohol exposure.

## Materials and methods

### Animals and experimental groups

FVB MMTV-Wnt1 [FVB.Cg-Tg (Wnt1)1Hev/J] transgenic and FVB *wild type* (*WT*) mice were obtained from The Jackson Laboratories (Bar Harbor, ME), bred, and housed in a climate-controlled animal facility. All procedures were reviewed and approved by the Institutional Animal Care and Use Committee (IACUC) of the University of Iowa. In this study, only female mice were used. Adolescent mice (5-week-old) or adult mice (10-week-old) from either FVB *wt* or MMTV-Wnt1 (Wnt1) transgenic strain were assigned into control and alcohol exposure groups. Two ages were selected because we previously demonstrated that adolescent Wnt1 mice were more susceptible to alcohol-induced mammary tumor promotion than adult mice (33). For alcohol exposure, the animals received a daily intraperitoneal (IP) injection of either PBS (control) or ethanol solution (2.5 g/kg, 25% w/v) for 15 days. The IP route was selected over oral gavage to model binge-like alcohol exposure while minimizing stress and gastrointestinal irritation, thereby allowing for more direct assessment of systemic ethanol effects on mammary tissue (33). All mice were monitored daily by palpation to ensure none developed mammary tumors.

**Figure 1:**
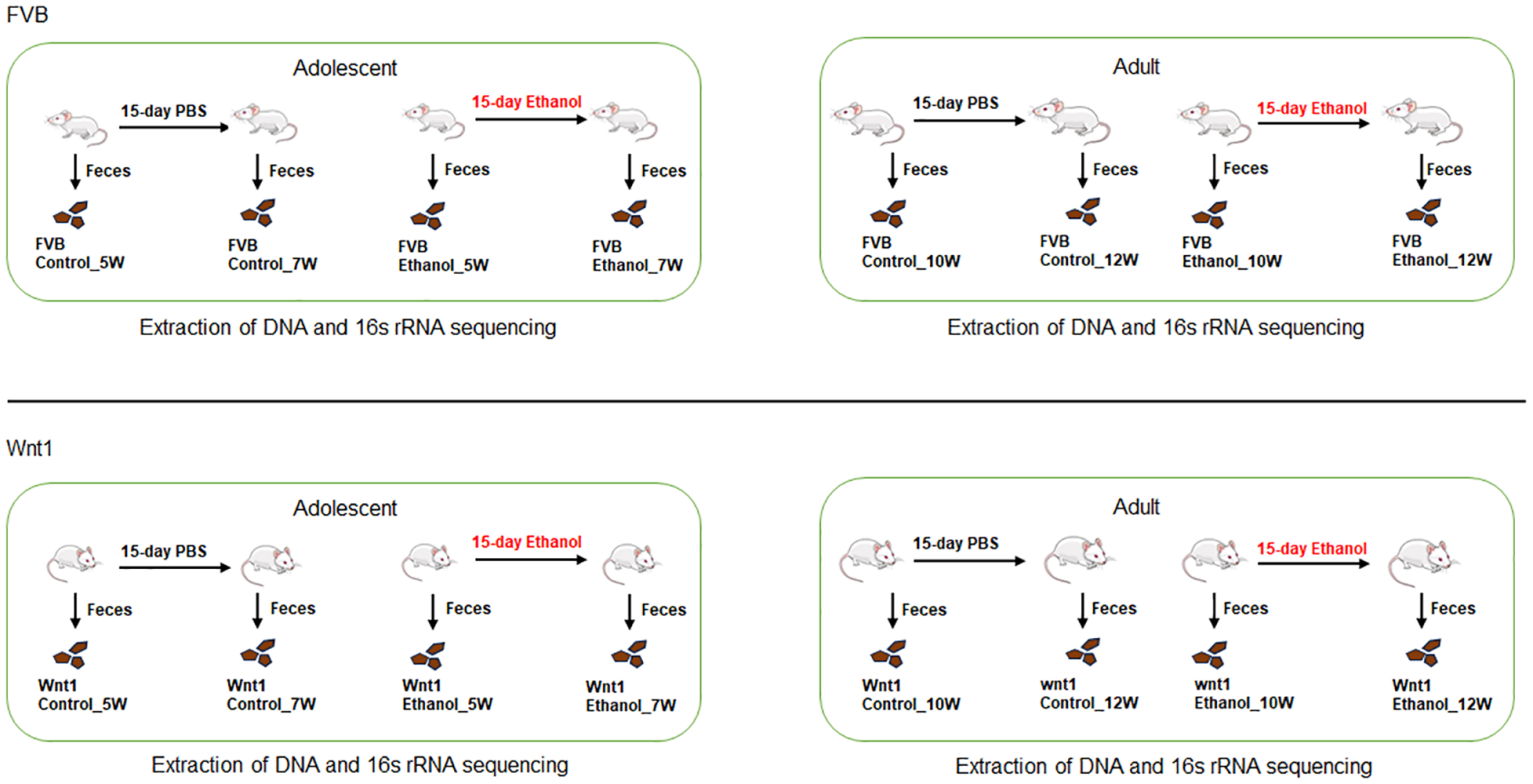
Design of experimental groups. Both adolescent (5-week-old) and adult (10-week-old) FVB and Wnt1 mice were treated with either PBS or ethanol for 15 days and one day after the treatment, their feces were collected and sent for DNA purification and 16s rRNA sequencing for gut microbiome composition. The number of each experimental groups are as follows: Adolescent FVB which includes control group before (FVB control_5W, n = 7) or after PBS treatment (FVB control_7W, n = 7) and ethanol group before (FVB Ethanol_5W, n = 5) or after ethanol treatment (FVB Ethanol_7W, n = 5); 2) Adult FVB including control group before (FVB control_10W, n = 8) or after PBS treatment (FVB control_12W, n = 8) and ethanol group before (FVB Ethanol_10W, n = 8) or after ethanol treatment (FVB Ethanol_12W, n = 8); 3) Adolescent Wnt1 which includes control group before (Wnt1 control_5W, n = 5) or after PBS treatment (Wnt1 control_7W, n = 5) and ethanol group before (Wnt1 Ethanol_5W, n = 7) or after ethanol treatment (Wnt1 Ethanol_7W, n = 7); 4) Adult Wnt1 including control group before (Wnt1 control_10W, n = 6) or after PBS treatment (Wnt1 control_12W, n = 6) and ethanol group before (Wnt1 Ethanol_10W, n = 7) or after ethanol treatment (Wnt1 Ethanol_12W, n = 7).

The experimental groups were assigned based on age, strain, and treatment (**Figure 1**). The adolescent mice started at 5 weeks old (5W) and became 7 weeks old (7W) after 15 days of treatment. The adult mice started at 10 weeks old (10W) and became 12 weeks old (12W) after a 15-day treatment period. There were four experimental groups of animals used in this study: 1) Adolescent FVB which included the control group before (Control_5W, n = 7) or after PBS treatment (Control_7W, n = 7) and the ethanol group before (Ethanol_5W, n = 5) or after ethanol treatment (Ethanol_7W, n = 5); 2) Adult FVB which included the control group before (Control_10W, n = 8) or after PBS treatment (Control_12W, n = 8) and the ethanol group before (Ethanol_10W, n = 8) or after ethanol treatment (Ethanol_12W, n = 8); 3) Adolescent Wnt1 which included the control group before (Control_5W, n = 5) or after PBS treatment (Control_7W, n = 5) and the ethanol group before (Ethanol_5W, n = 7) or after ethanol treatment (Ethanol_7W, n = 7); 4) Adult Wnt1 which included the control group before (Control_10W, n = 6) or after PBS treatment (Control_12W, n = 6) and the ethanol group before (Ethanol_10W, n = 7) or after ethanol treatment (Ethanol_12W, n = 7).

### Fecal sample collection, extraction of DNA and 16s RNA sequencing

Fecal samples from each animal were collected either one day before or after the exposure and stored at -80°C freezer until further processing for DNA extraction. Microbial DNA extraction, 16S rRNA amplicon, and sequencing were performed according to a previously published protocol (34). Briefly, DNA was isolated using DNeasy PowerLyzer PowerSoil Kit (Qiagen, Germantown, MD) as per the manufacturer’s instructions, including the recommended bead-beating step. The sequencing library was prepared using a 2-step amplification, where the V3-V4 region of the bacterial 16S rRNA gene was amplified in step 1, and barcodes were added using the Nextera XT Index Kit (Illumina MiSeq) in step 2. PCR products were purified and sequenced using the Illumina MiSeq platform. The R based platform Divisive Amplicon Denoising Algorithm 2 (DADA2) (35) was used to trim, merge, and filter reads and generate an amplicon sequence variant (ASV) table. The ASVs were taxonomically classified from kingdom to species levels using the Silva database (version 138.1), with a median read count of 49,839 (ranging from 1,667 to 78,455 reads).

### Microbiome analyses and visualization

Microbial communities were analyzed using previously described methods for each experimental group (30, 34, 36). Briefly, custom R (Version 4.3.1) scripts were utilized, integrating packages such as phyloseq (37), vegan (38), ggpubr (39), dplyr (40), microbiome (41), tidyr (42), sigminer (43), and ggplot2 (44). Except for alpha diversity, reads underwent normalization using constant-sum scaling and log10 transformation at the bacterial level to their median sequencing depth. Alpha diversity analysis was conducted on unfiltered data using the Chao1 index. Beta diversity was assessed via Principal Component Analysis (PCA) based on weighted UniFrac distances, with significance tested through PERMANOVA. A heatmap of the most abundant genera was generated using the phyloseq (37) and ggplot2 (44) packages, visualizing top bacterial genera based on weighted UniFrac distances (45). Multidimensional Scaling (MDS) was employed for ordination, with sample groups arranged along the x-axis to represent relative abundance. To visualize enrichment, the LEfSe plot was produced using the microbiomeMarker (46) package’s “run_lefse” function, highlighting the genera enriched in the different groups within each experimental group using the Kruskal-Wallis test.

### Statistical analyses

A two-way analysis of variance (ANOVA) was conducted with age and treatment as independent variables to analyze the relative abundance of selected microbial taxa across experimental groups. *Post hoc* comparisons were performed using Tukey’s correction for multiple comparisons, utilizing GraphPad Prism Version 10.3.1 (GraphPad Software, Inc., www.graphpad.com). Additionally, the Wilcoxon matched-pairs signed rank test was applied to assess the effects of alcohol on the abundance of microbial taxa in selected treatment groups. A significance threshold of p < 0.05 was set for all analyses.

## Results

### Microbiome analysis of alpha diversity of alcohol-exposed mice

**Figure 2:**
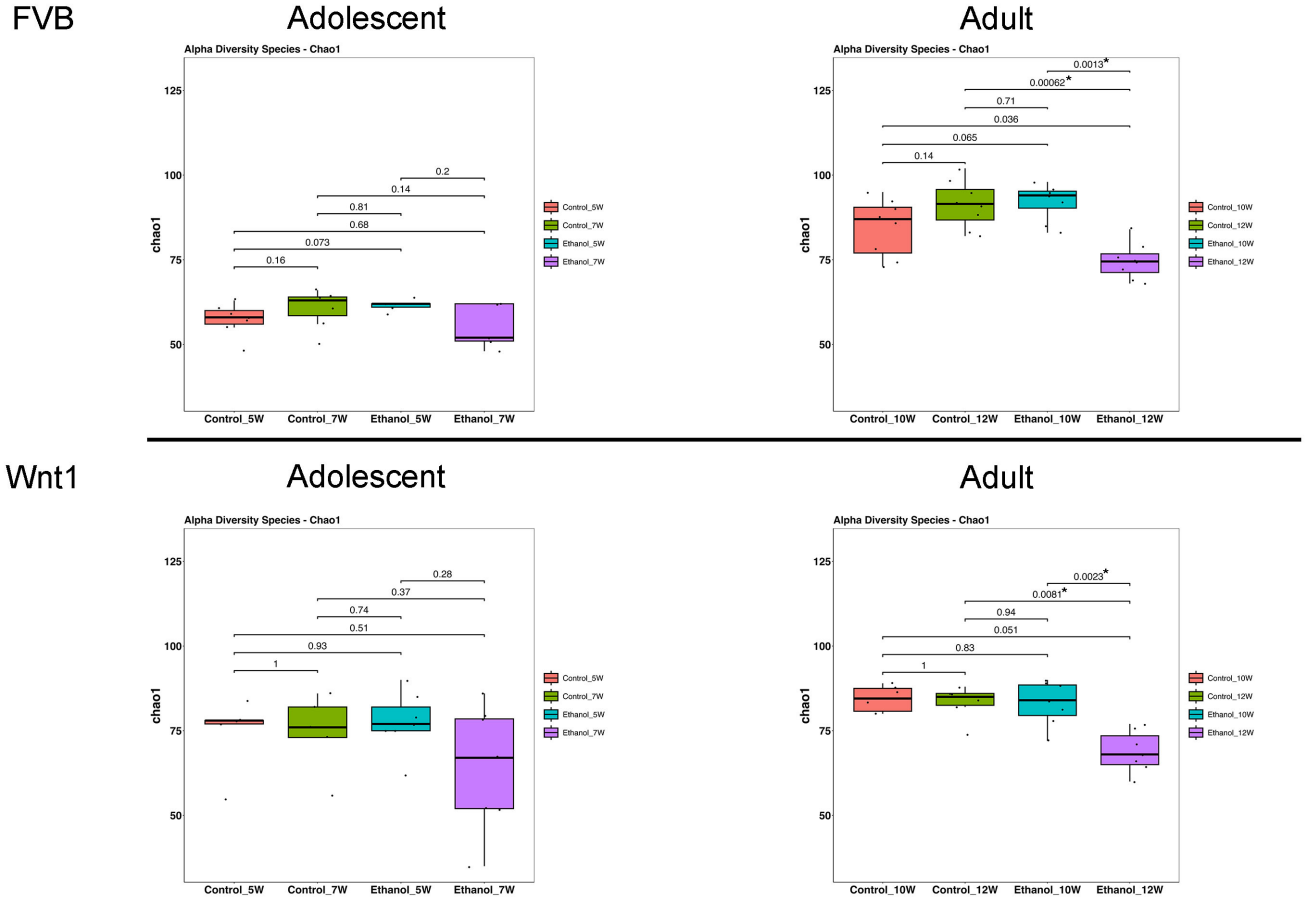
Effects of alcohol on microbial species richness. The effect of alcohol on alpha diversity was measured by Chao1 index in all experimental groups: Overall group differences were analyzed using the Kruskal–Wallis test, followed by pairwise comparisons with Wilcoxon rank-sum tests. Alcohol exposure significantly reduced alpha diversity in adult mice. *p < 0.05.

Alpha diversity refers to within-sample diversity. When examining alpha diversity, we are able to evaluate the distribution of microbes within a sample or metadata category. The Chao1 index, a statistical estimator that measures species richness, is widely used to assess alpha diversity in microbiome research including gut microbiome (47). We used Chao1 index to determine the effect of alcohol exposure on alpha diversity (**Figure 2**). In FVB mice, there was no significant difference in the Chao1 index between control groups in either adolescents (p _control_7W vs. control_5W_ = 0.16) or adults (p_control_12W vs. control_10W_ = 0.14). Alcohol exposure did not significantly change the Chao1 index in adolescent mice (p_Ethanol_7W vs. Ethanol_5W_ = 0.2), but significantly reduced the number of microbial species in adults (p_Ethanol_12W vs. Ethanol_10W_ = 0.0013). In Wnt1 mice, the Chao1 index similarly showed no significant differences between control groups either in adolescents (p_control_7W vs. control_5W_ = 1) or adults (p_control_12W vs. control_10W_ = 1). Alcohol exposure did not significantly alter the Chao1 index in adolescents (p_Ethanol_7W vs. Ethanol_5W_ = 0.28), but significantly reduced microbial richness in adults (p_Ethanol_12W vs. Ethanol_10W_ = 0.0023). These findings demonstrate that alcohol exposure significantly reduced alpha diversity in adults in both mouse strains, whereas the impact on the adolescents was not significant.

### Microbiome analysis of beta diversity of alcohol-exposed mice

**Figure 3:**
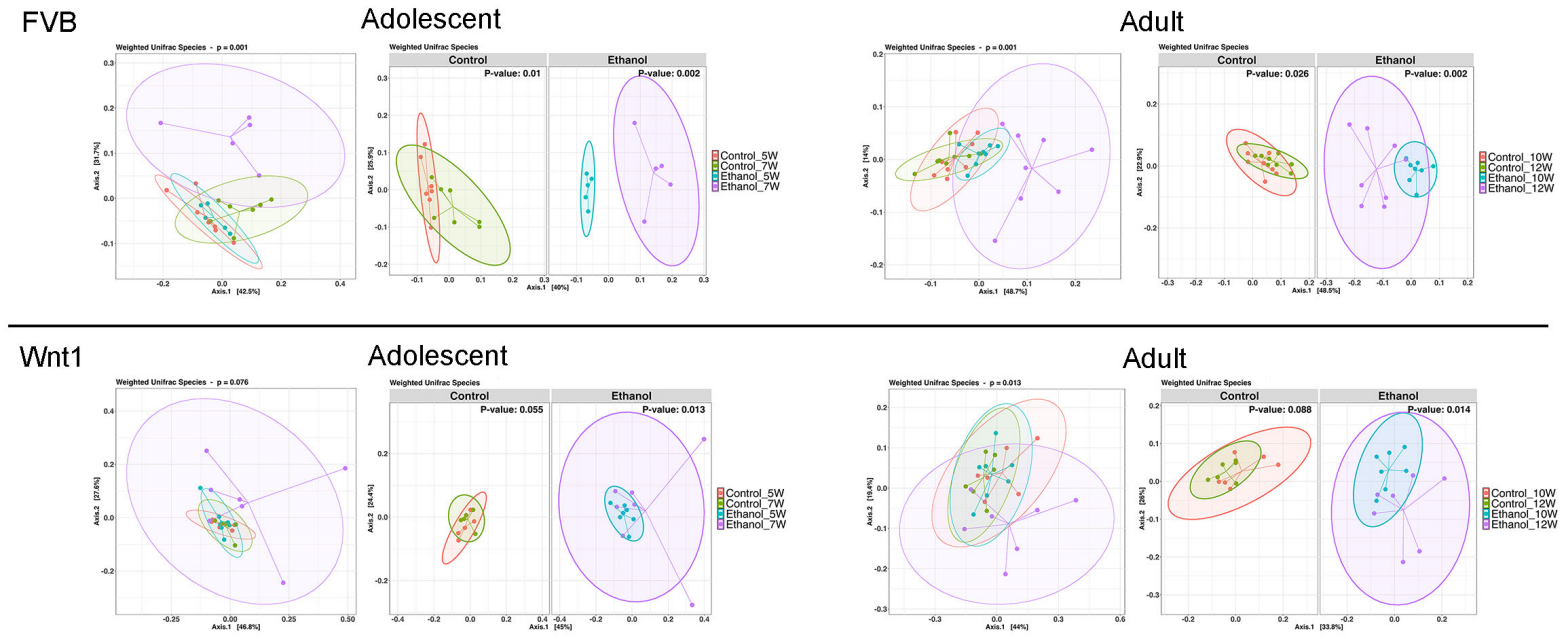
Effects of alcohol on microbial compositions. The effect of alcohol on beta diversity was determined by weighted UniFrac analysis in all experimental groups. PERMANOVA (adonis2) was applied to test for differences between groups. Alcohol exposure significantly changed the microbial compositions in adolescents and adults in both strains. The percentages shown on Axis.1 and Axis.2 represent the proportion of total variation in the microbial community composition that is captured by each principal coordinate. For instance, a label of “42.5%” on Axis.1 indicates that this axis explains 42.5% of the variation in the pairwise Weighted UniFrac distances among samples. Similarly, “31.7%” on Axis.2 represents an additional 31.7% of variation explained.

Beta diversity is the diversity between samples and a common statistical method to assess the similarity or differences in microbial compositions between samples (47). To examine the impact of alcohol exposure on microbial diversity across experimental groups, we employed weighted UniFrac, a quantitative measure of beta diversity (**Figure 3**). In FVB mice, treatment significantly affected gut microbial composition in both adolescents (p = 0.001) and adults (p = 0.001). Further analysis by treatment revealed significant differences in the microbial community in controls across age groups for both adolescents (p_control_7W vs. control_5W_ = 0.01) and adults (p_control_12W vs. control_10W_ = 0.025), and an even more marked differences in alcohol groups in both adolescents (p_Ethanol_7W vs. Ethanol_5W_ = 0.002) and adults (p_Ethanol_12W vs. Ethanol_10W_ = 0.002). A similar trend was observed in Wnt1 mice, with a near-significant treatment effect on gut microbial composition in adolescents (p = 0.076), and a significant effect in adults (p = 0.013). When separated by treatment, the control groups showed a nearly significant difference in adolescents (p_control_7W vs. control_5W_ = 0.055) and adults (p_control_12W vs. control_10W_ = 0.088), while significant differences were observed in alcohol-exposed adolescents (p_Ethanol_7W vs. Ethanol_5W_ = 0.013) and adults (p_Ethanol_12W vs. Ethanol_10W_ = 0.014). These results suggested that alcohol exposure has a more pronounced impact on the beta diversity, compared to the effects with natural age-related development in controls regardless of strains and ages.

### The abundance of microbial populations of alcohol-exposed mice

**Figure 4:**
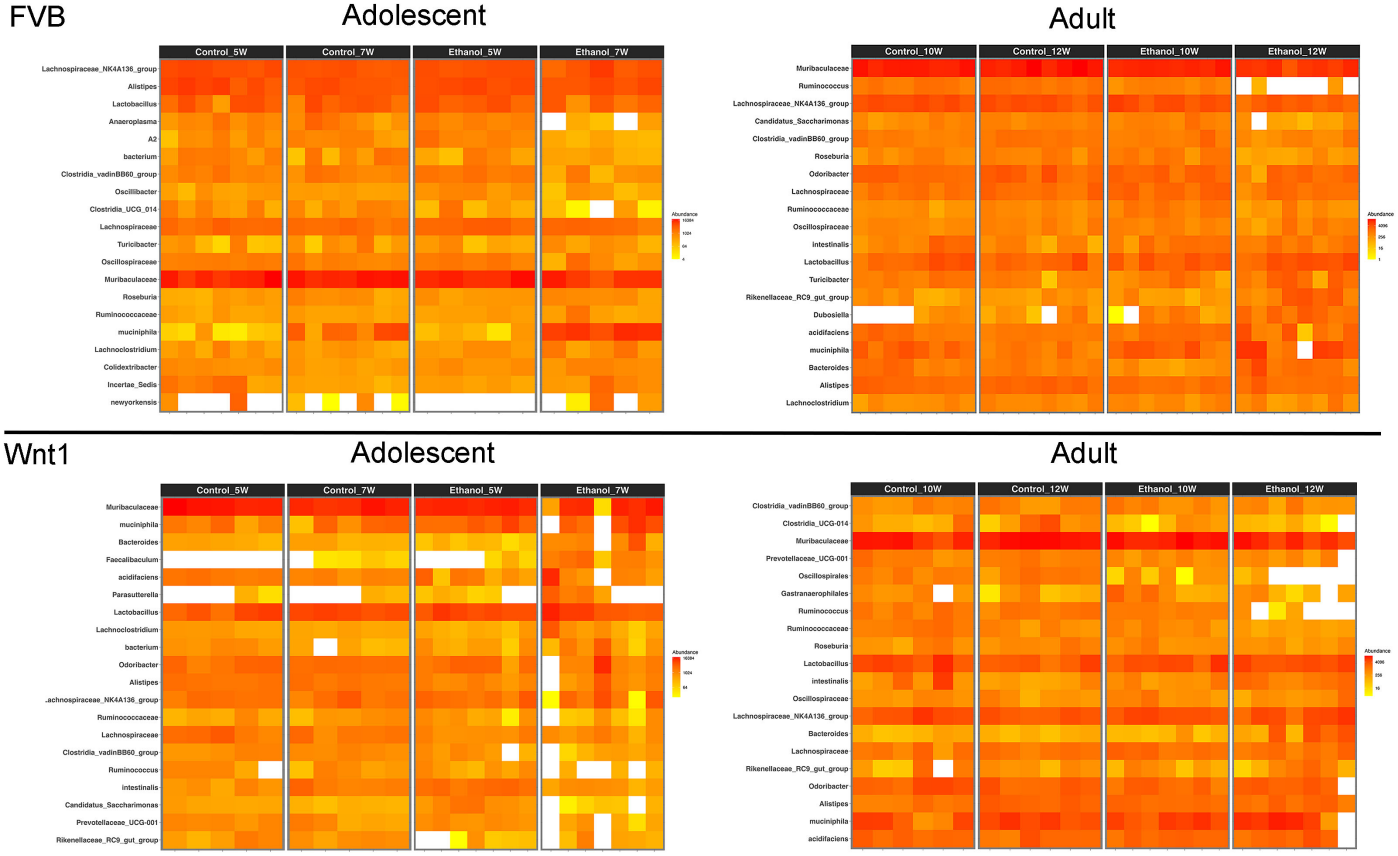
Effects of alcohol on microbial taxa. Heat maps show the top 20 most prevalent microbial taxa within each experimental group. Genus names have been used to represent taxa, including *Lachnospiraceae_NK4A136_group* (for *bacterium*), *Dubosiella* (for *newyorkensis*), *Bacteroides* (for *acidifaciens*), *Akkermansia* (for *muciniphila*), *Lactobacillus* (for *intestinalis*), and *Lachnospiraceae* (for *A2*). Full taxonomic classifications can be accessed in the 16S rRNA gene sequencing data deposited in the Sequence Read Archive (SRA) under BioProject ID: PRJNA1248563.

To highlight the most abundant microbial taxa in each experimental group, we used heatmap visualization to display the top 20 most prevalent microbial populations (**Figure 4**). The heatmap analysis revealed a similar pattern of microbial abundance across the experimental groups. In both adolescent and adult FVB experimental groups, the commonly identified taxa included the species *Akkermansia muciniphila*; the genera *Turicibacter*, *Lachnoclostridium*, *Lactobacillus*, *Lachnospiraceae_NK4A136_group*, and *Alistipes*; the families *Muribaculaceae*, *Oscillospiraceae*, and *Ruminococcaceae*; and the order *Clostridia_vadinBB60_group*. In the adolescent and adult Wnt1 experimental groups, the taxa commonly identified were the species *A. muciniphila* and *Lactobacillus intestinalis*; the genera *Bacteroides*, *Lachnospiraceae_NK4A136_group*, *Lactobacillus*, *Prevotellaceae_UCG-001*, *Alistipes*, *Rikenellaceae_RC9_gut_group*, and *Ruminococcus*; the families *Lachnospiraceae* and *Ruminococcaceae*. The analysis showed distinct yet overlapping microbial profiles across experimental groups, with certain taxa appearing as key microbial populations such as *A. muciniphila*, and members of *Lachnospiraceae* and *Ruminococcaceae* families, present in both FVB and Wnt1 groups. This combination of both shared and distinct microbial populations between the FVB and Wnt1 groups may relate to their age and genetic backgrounds.

### The differential microbial populations of alcohol-exposed mice

**Figure 5:**
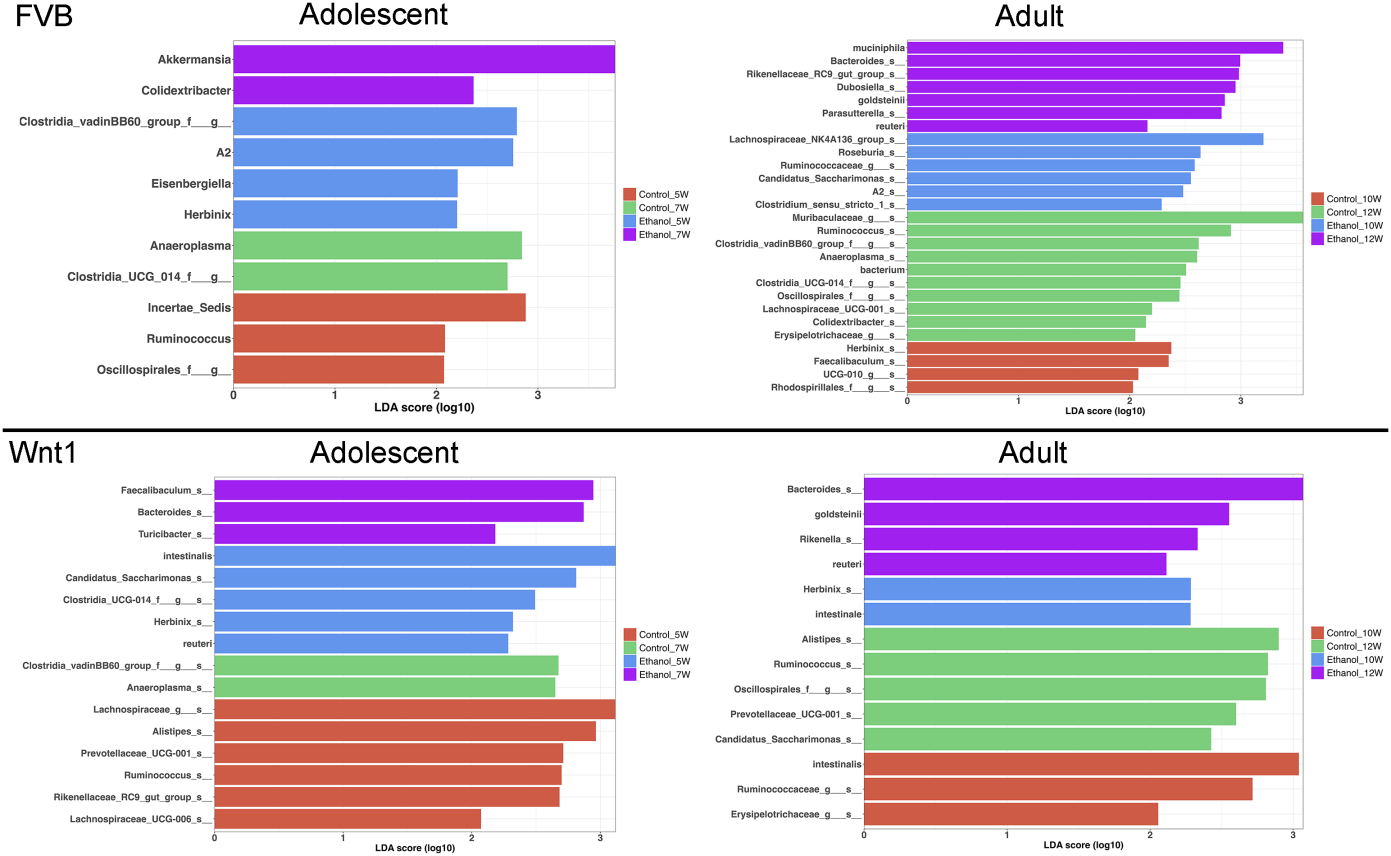
Effects of alcohol on specific microbial taxa. Linear Discriminant Analysis Effect Size (LEfSe) analysis was performed to identify the microbial taxa that were mostly affected by alcohol exposure.

We then performed Linear Discriminant Analysis Effect Size (LEfSe) analysis to identify the microbial populations that are most affected by alcohol exposure in each experimental group (**Figure 5**). LEfSe is a biomarker discovery tool that identifies statistically significant differences between multiple groups. In the adolescent FVB experimental group, LEfSe analysis revealed that the species *A. muciniphila* and the genus *Colidextribacter* were the most enriched taxa in alcohol-exposed mice. In the adult FVB experimental group, alcohol exposure was associated with an enrichment of the species *A. muciniphila*, *Lactobacillus reuteri*, and *Parabacteroides goldsteinii*, as well as the genera *Bacteroides*, *Dubosiella*, *Rikenellaceae_RC9_gut_group*, and *Parasutterella*. In the adolescent Wnt1 experimental group, alcohol exposure led to a higher abundance of the genera *Faecalibaculum*, *Bacteroides*, and *Turicibacter*. In the adult Wnt1 experimental group, alcohol exposure was associated with an enrichment of the species *P. goldsteinii* and *L. reuteri*, along with the genera *Bacteroides* and *Rikenella*. These results indicate that age and genetic background significantly affect the microbiome’s response to alcohol, with both overlapping and unique taxa affected across experimental groups.

### Comparisons of taxonomic abundance of selective microbial population

We conducted a two-way analysis of variance (ANOVA) for each microbial taxon that was identified in the LEfSe analysis to validate the findings in each experimental group (**Supplementary Figures 4**-**7**). We further analyzed microbial taxa that were significantly altered by alcohol exposure in adolescent Wnt1 mice, because they were more sensitive to alcohol’s tumor promotion compared to adults (33). We also compared the relative abundance of specific taxa across other experimental groups.

**Figure 6:**
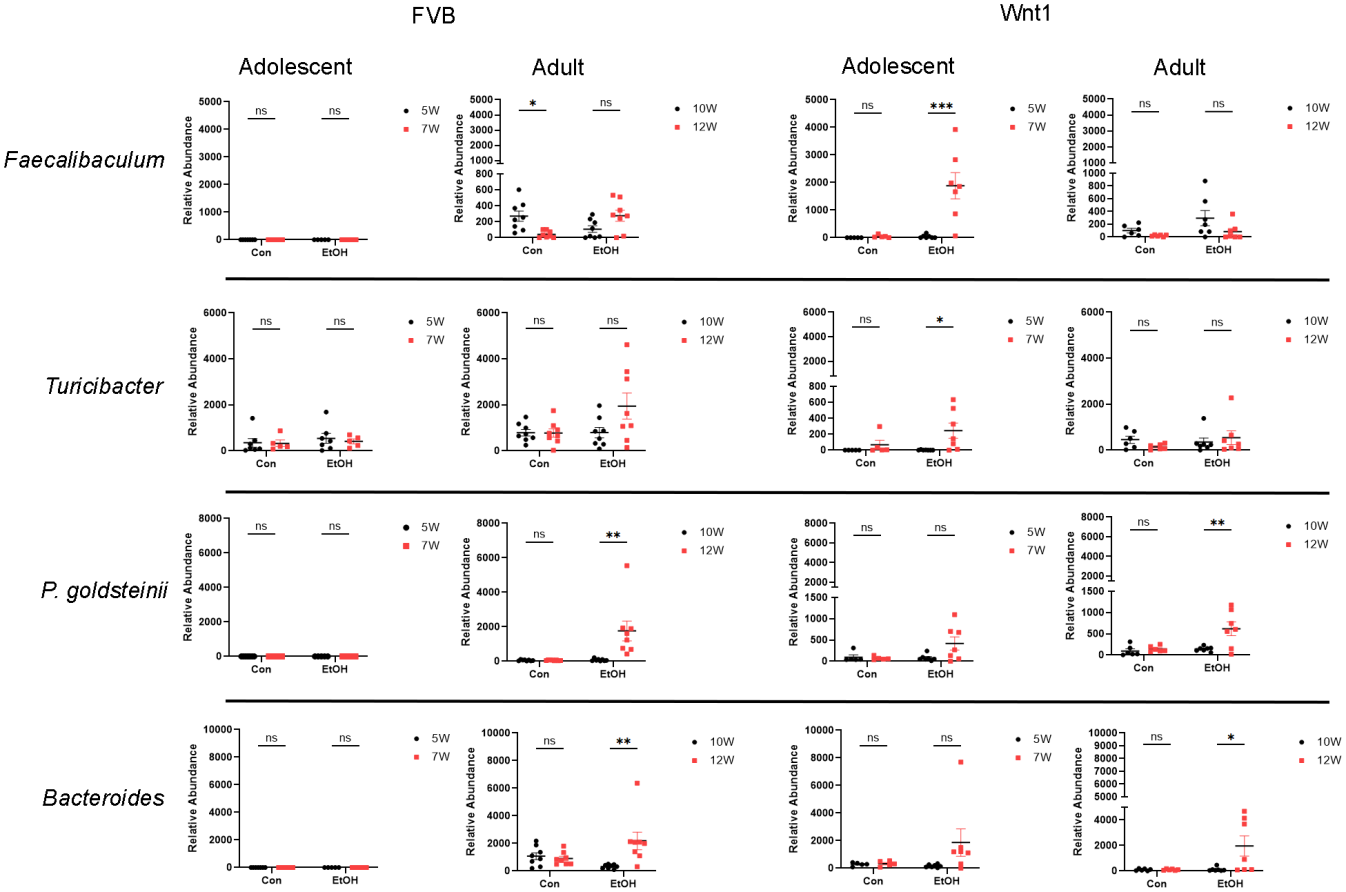
Analysis of alcohol-enriched microbial taxa. Alcohol-enriched microbial taxa were analyzed by two-way ANOVA followed by Tukey’s *post hoc* test. Selective microbial taxa including the species *P. goldsteinii*; the genera *Faecalibaculum*, *Turicibacter* and *Bacteroides* were increased by alcohol exposure. FVB: n = 7 for control (Con) 5W and 7W; n = 5 for ethanol (EtOH) 5W and 7W; n = 8 for control (Con) 10W and 12W; n = 8 for ethanol (EtOH) 10W and 12W; Wnt1: n = 5 for control (Con) 5W and 7W; n = 7 for ethanol (EtOH) 5W and 7W; n = 6 for control (Con) 10W and 12W; n = 7 for ethanol (EtOH) 10W and 12W. ∗p < 0.05, ∗∗p < 0.01, ∗∗∗p < 0.001, ns = not significant (p > 0.05).

Alcohol exposure increased the abundance of several microbial taxa in the adolescent Wnt1 experimental group, including *P. goldsteinii* and the genera *Bacteroides, Faecalibaculum*, and *Turicibacter*. The abundance of these taxa was analyzed across various experimental groups using two-way ANOVA followed by Tukey’s *post hoc* test (**Figure 6**). Levels of *P. goldsteinii* showed a nearly significant increase in alcohol-exposed adolescent Wnt1 mice (p = 0.062) and were significantly enriched in adult Wnt1 and adult FVB mice. Although *Bacteroides* levels showed a non-significant increase in the adolescent Wnt1 experimental group by two-way ANOVA followed by Tukey’s *post hoc* test (p = 0.1590), the Wilcoxon test revealed a significant effect (p = 0.0469, **Supplementary Figure 1**). Both adult Wnt1 and FVB experimental groups exhibited a significant increase in *Bacteroides* following alcohol exposure. *Faecalibaculum* levels were significantly elevated in alcohol-exposed adolescent Wnt1 mice but were notably decreased in the control group of adult FVB mice. Levels of *Turicibacter* were significantly increased in the adolescent Wnt1 experimental group than that in the control group, with a near-significant increase also observed in the adult FVB experimental group (p = 0.0801).

Although *A. muciniphila* did not show a significant increase in alcohol-exposed adolescent Wnt1 mice, it is one of the most abundant bacterial species in the gut microbiome and was identified in both the heatmap and LEfSe analysis across multiple experimental groups (**Supplementary Figure 2**). A detailed examination revealed that *A. muciniphila* was significantly elevated by alcohol exposure only in the adolescent FVB group, with no significant changes observed in other groups.

**Figure 7:**
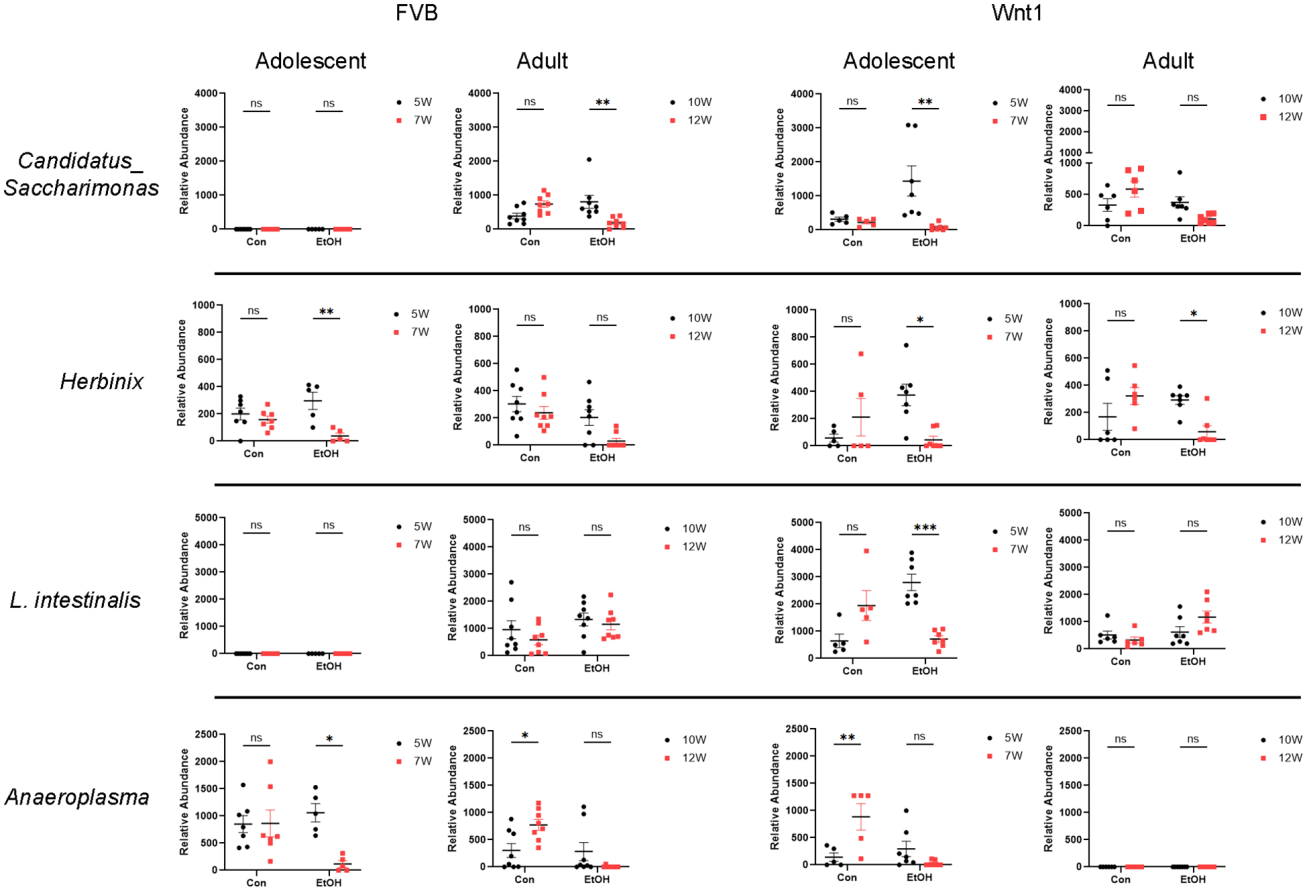
Analysis of alcohol-depleted microbial taxa. Alcohol-depleted microbial taxa were analyzed by two-way ANOVA followed by Tukey’s *post hoc* test. Selective microbial taxa such as the species *L. intestinalis* and the genera *Candidatus_Saccharimonas*, *Herbinix* and *Anaeroplasma* were reduced by alcohol exposure. FVB: n = 7 for control (Con) 5W and 7W; n = 5 for ethanol (EtOH) 5W and 7W; n = 8 for control (Con) 10W and 12W; n = 8 for ethanol (EtOH) 10W and 12W; Wnt1: n = 5 for control (Con) 5W and 7W; n = 7 for ethanol (EtOH) 5W and 7W; n = 6 for control (Con) 10W and 12W; n = 7 for ethanol (EtOH) 10W and 12W. ∗p < 0.05, ∗∗p < 0.01, ∗∗∗p < 0.001, ns, not significant (p > 0.05).

In contrast, the abundance of certain microbial taxa, including the species *L. intestinalis* and the genera *Anaeroplasma*, *Herbinix*, and *Candidatus_Saccharimonas*, was decreased following alcohol exposure in the adolescent Wnt1 experimental group, with further analysis across all experimental groups (**Figure 7**). *L. intestinalis* was significantly reduced in the alcohol-exposed adolescent Wnt1 experimental group, with no similar effects observed in other experimental groups. In the same experimental group, the *Anaeroplasma* level was significantly higher in control mice but lower in the alcohol-exposed mice, although this reduction was not statistically significant (p = 0.4502). The Wilcoxon test, however, showed a significant effect of alcohol on *Anaeroplasma* (p = 0.0312, **Supplementary Figure 1**). In addition, *Anaeroplasma* was significantly reduced following alcohol exposure in the adolescent FVB experimental group. *Herbinix* exhibited a consistent decrease following alcohol exposure across the adolescent Wnt1, adult Wnt1, and adolescent FVB experimental groups, with a nearly significant reduction in the adult FVB experimental group (p = 0.0715). Finally, *Candidatus_Saccharimonas* level was decreased in response to alcohol exposure in both the adolescent Wnt1 and adult FVB experimental groups.

## Discussion

The Wnt1 transgene, driven by the mouse mammary tumor virus (MMTV) promoter, is a well-established approach for development of a mouse model to study mammary tumor development, as the Wnt1 signaling pathway is crucial for regulating cell proliferation, differentiation and development (48, 49). We previously employed this model to investigate alcohol-induced tumor promotion and found that daily intraperitoneal (IP) injections of ethanol (2.5 g/kg, 25% w/v) for 15 days significantly shortened tumor onset, increased lung metastasis, and elevated circulating levels of estradiol and progesterone, with adolescent mice showing greater sensitivity to the effects of alcohol than adults (33). This 15-day ethanol exposure paradigm was designed to mimic early-stage, heavy episodic alcohol consumption — a pattern prevalent among adolescents and young adults in the U.S. and Europe (50–53) and is more common among females in the U.S. (54). In this study, we further utilized this model to examine the impact of alcohol on the gut microbiome. Our results demonstrated that alcohol exposure significantly reduced microbial species richness as indicated by decreased Chao1 index in adult mice, while it had little effects on this index in adolescent mice. Alcohol also altered microbial compositions as indicated by the analysis of Beta diversity in both adolescents and adults in both strains. Comparative profiling identified a number of taxa consistently affected by alcohol across groups. Further LEfSe and two-way ANOVA analyses confirmed that specific taxa were targets of alcohol exposure.

We first examined the impact of alcohol exposure on alpha diversity by assessing species richness with the Chao1 index (**Figure 2**) and species diversity with the Shannon index (**Supplementary Figure 3**). While alcohol exposure did not significantly alter the Shannon index, it had a notable impact on the Chao1 index. A reduced Chao1 index indicating a lower alpha diversity in the gut microbiome, has been reported in patients with breast cancer when compared to healthy controls in multiple studies (55–59). However, there are studies showing that breast cancer patients have different microbial compositions without any difference in alpha diversity (30, 60, 61). An intriguing finding from our study is that the Chao1 index in adolescent mice appeared more resilient to the adverse effects of alcohol exposure compared to adults (**Figure 2**), suggesting greater plasticity of the adolescent gut microbiome. The microbiome plasticity, or the ability of the gut microbiome to adapt to environmental changes, is known to be highest early in life and decline with age (62–64). Alternatively, this differential response may reflect age-related differences in hepatic alcohol metabolism, where adolescent mice exhibit higher alcohol dehydrogenase and lower aldehyde dehydrogenase activity, potentially limiting acetaldehyde accumulation and associated microbial disruption (65). Together, these findings suggest that while adolescents may be more vulnerable to alcohol’s systemic effects in the context of breast cancer, their gut microbiota may retain greater adaptive capacity, resulting in a more nuanced and multifactorial response to alcohol exposure.

Our beta diversity analysis using weighted UniFrac showed that changes in microbial communities over time were more pronounced in Wnt1 mice compared to FVB mice, with significant alterations observed in the alcohol-exposed groups relative to controls. These results suggest that alcohol exposure significantly disrupts the beta diversity of the gut microbiome over time, beyond the natural developmental changes observed in controls, and Wnt1 mice were more sensitive to alcohol-induced alterations. The increased sensitivity of gut microbiota in Wnt1 mice, may be attributed to the role of Wnt1 signaling in regulating cell proliferation and differentiation, which could interact with the gut microbiome, rendering it more susceptible to environmental factors such as alcohol. Notably, altered beta diversity in the gut microbiome has been linked to various stages of breast cancer, including its subtypes and the presence of metastatic disease (28, 55, 66, 67). These variations in microbial diversity might correlate with systemic inflammation or immune markers, potentially influencing tumor progression and responses to therapies such as chemotherapy or hormone therapy (68, 69).

Comparative analysis of microbial profiles, visualized as heatmaps, revealed distinct yet overlapping responses to alcohol exposure across experimental groups in both FVB and Wnt1 mice. The commonly identified taxa in all experimental groups included the species *A. muciniphila*; the genera *Lachnospiraceae_NK4A136_group*, *Lactobacillus* and *Alistipes*; and the family *Ruminococcaceae. A. muciniphila* is a mucus-degrading bacterium that resides in the gut’s mucus layer and is often associated with gut barrier integrity and metabolic health (70, 71). The genus *Lachnospiraceae_NK4A136_group*, part of the family of *Lachnospiraceae*, is known for producing short-chain fatty acids (SCFAs) such as butyrate, which supports gut health (19, 20). The genus *Lactobacillus* comprises numerous probiotic species known for their ability to produce lactic acid and promote gut health (72). Additionally, *Alistipes*, from the *Bacteroidetes* phylum, is often linked with protein fermentation and bile acid metabolism (73). The *Ruminococcaceae* family plays a role in fiber degradation and SCFA production, including butyrate, which supports gut barrier function (74). Although Wnt1 signaling can affect the gut microbiome through its effects on the gut environment and epithelial cell turnover (75, 76), the similar microbial profiles observed in both FVB and Wnt1 mice suggest that alcohol exposure exerts a universal impact on the gut microbiome composition. The combination of shared and unique microbial populations across FVB and Wnt1 groups may reflect variations in age and genetic backgrounds.

To identify specific microbial taxa that were significantly impacted by alcohol exposure, we performed LEfSe and two-way ANOVA analysis. These analyses revealed that a number of selective microbial taxa were increased following alcohol exposure; they include the genera *Bacteroides*, *Faecalibaculum* and *Turicibacter*, the species *P. goldsteinii* and *A. muciniphila*. The genus *Bacteroides* is among the most abundant genera in the gut, including species that play diverse roles ranging from beneficial to pathogenic (77). For example, *Bacteroides fragilis*, often isolated from extra-intestinal infections, can cause inflammation when it translocates from the gut to other organs due to a compromised intestinal barrier (78–80). *Bacteroides fragilis* and other *Bacteroides* species such as *Bacteroides uniformis* and *Bacteroides vulgatus* are known to produce beta-glucuronidase, an enzyme involved in the metabolism of estrogens and may influence estrogen-sensitive breast cancer (81, 82). Interestingly, *Bacteroides fragilis* has also been shown to exert anti-cancer and anti-proliferative effects in mouse breast cancer models (83). The genus *Faecalibaculum*, from the *Erysipelotrichaceae* family, plays a critical role in the anti-tumor effects of combined therapies using anti-PD-1 antibody and dietary supplement fucoidan in a breast cancer mouse model (84). One of the *Faecalibaculum* species, *Faecalibaculum rodentium*, originally identified as anti-tumorigenic in a mouse model for colorectal cancer (85), was shown to counteract the antibiotic-induced tumor growth acceleration in multiple breast cancer mouse models (86). Meanwhile, *Turicibacter*, typically present at low or moderate levels in the gut, is involved in the metabolism of lipids and bile acids (87). However, higher levels of *Turicibacter* have been detected in the intra-tumoral microbiome of patients with triple-negative breast cancer (TNBC) (88) and in the gut microbiota of premenopausal breast cancer patients (89). *P. goldsteinii* is a low-abundant probiotic in the gut microbiome that supports intestinal integrity and reduces inflammation in conditions such as obesity (90) and pulmonary diseases (91). Finally, *A. muciniphila*, a mucin-degrading bacterium with anti-inflammatory properties, has been associated with improved outcomes in metabolic disorders, intestinal inflammation and several cancers (92).

The LEfSe and two-way ANOVA analyses also revealed that alcohol exposure led to the reduction of several key microbial taxa: the species *L. intestinalis*, the genera *Candidatus Saccharimonas*, *Herbinix* and *Anaeroplasma*. *L. intestinalis* is a known probiotic that has been shown to support gut health and immune regulation in a variety of disease models (93–95). *Candidatus Saccharimonas*, a genus within the family *Candidatus Saccharimonadaceae*, has been identified primarily through genetic analysis but remains uncultured, therefore further research is needed to elucidate its functional role. *Herbinix*, though less studied than other genera in the *Lachnospiraceae* family, is part of a group known for SCFA production, which plays a crucial role in maintaining gut health. This genus is frequently identified in microbiome studies through sequencing data and contributes to the overall microbial composition of the gut (96, 97). *Anaeroplasma* has been shown to exhibit anti-inflammatory effects in lung diseases (98) and improve bile acid metabolism and enhance gut barrier function in metabolic disorders (99, 100). Nonetheless, *Anaeroplasma* has been negatively correlated with the efficacy of naringenin, a flavonoid compound in citrus fruits, in the treatment of non‐alcoholic fatty liver disease (101).

In summary, our findings reveal that even short-term alcohol exposure significantly reduces gut microbiome diversity and disrupts specific microbial communities (**Supplementary Table 1**). These alterations are biologically meaningful and may persist beyond the exposure period. For instance, Llopis et al. (102) showed that two weeks of alcohol feeding altered the gut microbiota, triggering systemic inflammation and increased intestinal permeability—effects that were reversed by microbiota transplantation, establishing a causal link between alcohol-induced dysbiosis and disease (102). Interestingly, we observed a more pronounced reduction in alpha diversity in adult mice compared to adolescents, a finding that contrasts with our previous work showing that adolescent mice are more susceptible to alcohol-induced mammary tumorigenesis (33). This seeming discrepancy highlights the complex and context-dependent role of the microbiota in cancer susceptibility, which may vary with developmental stage, immune status, and intrinsic tumor risk.

The specific microbial taxa altered in this study such as *Turicibacter*, *Faecalibaculum*, *P. goldsteinii*, *L. intestinalis*, and *A. muciniphila* (**Supplementary Table 1**), are known to regulate inflammation, a critical factor in carcinogenesis. Moreover, *Bacteroides fragilis* and other species, including *Bacteroides uniformis* and *Bacteroides vulgatus*, are implicated in estrogen metabolism, potentially influencing hormone-driven breast cancer pathways. While it remains to be determined whether these observed microbial shifts are causative or merely correlative, functional validation experiments such as fecal microbiota transplantation could clarify their mechanistic role. It is also currently unclear whether the alcohol-induced alterations in microbiome are reversible or not. A future study examining the effects of alcohol after a prolonged abstinence may be necessary. Regardless it is reversible or persistent, our results reveal that alcohol can impact microbiome in a preclinical model of breast cancer development.

We acknowledge there are some limitations in this study. First, microbial abundance was not independently validated; complementary methods such as qPCR or targeted bacterial cultures would strengthen these findings. Second, we administered alcohol via intraperitoneal (IP) injection to model binge-like exposure with controlled dosing and to minimize stress and gastrointestinal irritation associated with oral gavage. While this approach is justified for this study, it does not fully replicate the physiological process of alcohol consumption through the gastrointestinal tract, which may differentially impact gut microbiome composition. Studies using oral gavage administration and appropriate control groups (e.g., handling or gavage controls) may provide additional insight into the effects of alcohol *consumption* on the gut microbiome.

Together, our results underscore the relevance of alcohol-induced gut dysbiosis in shaping the tumor microenvironment and lay a foundation for future research into microbiota-targeted strategies for breast cancer prevention and therapy.

## Data Availability

The 16S rRNA gene sequencing raw data generated and/or analyzed during this study have been uploaded to the Sequence Read Archive (SRA) under the BioProject ID: PRJNA1248563 for free public access.

## Supplementary material

Supplementary Figure 1 Wilcoxon matched-pairs signed rank test was applied to assess the effects of alcohol exposure on *Bacteroides* in adolescent Wnt1 mice (left), *Anaeroplasma* in adolescent Wnt1 mice (right). ∗p < 0.05.

Supplementary Figure 2 The effects of alcohol exposure on the species *A. muciniphila*. Two-way ANOVA followed by Tukey’s *post hoc* test was applied to analyze *A. muciniphila* in both adolescents (left) and adults (right) in FVB (left panel) and Wnt1 mice (right panel). FVB: n = 7 for control (Con) 5W and 7W; n = 5 for ethanol (EtOH) 5W and 7W; n = 8 for control (Con) 10W and 12W; n = 8 for ethanol (EtOH) 10W and 12W; Wnt1: n = 5 for control (Con) 5W and 7W; n = 7 for ethanol (EtOH) 5W and 7W; n = 6 for control (Con) 10W and 12W; n = 7 for ethanol (EtOH) 10W and 12W. ∗p < 0.05, ∗∗p < 0.01, ∗∗∗p < 0.001, ∗∗∗∗p < 0.0001, ns = not significant (p > 0.05)

Supplementary Figure 3 Effects of alcohol on microbial species richness. The effect of alcohol on alpha diversity was also measured by Shannon index in all experimental groups.

Supplementary Figure 4 Two-way ANOVA followed by Tukey’s *post hoc* test for each microbial taxon identified in the LEfSe analysis of adolescent FVB mice. ∗p < 0.05, ∗∗p < 0.01, ∗∗∗p < 0.001, ns = not significant (p > 0.05).

Supplementary Figure 5 Two-way ANOVA followed by Tukey’s *post hoc* test for each microbial taxon identified in the LEfSe analysis of adult FVB mice **(A, B)**. ∗p < 0.05, ∗∗p < 0.01, ∗∗∗p < 0.001, ns = not significant (p > 0.05).

Supplementary Figure 6 Two-way ANOVA followed by Tukey’s *post hoc* test for each microbial taxon identified in the LEfSe analysis of adolescent Wnt1 mice. ∗p < 0.05, ∗∗p < 0.01, ∗∗∗p < 0.001, ns = not significant (p > 0.05).

Supplementary Figure 7 Two-way ANOVA followed by Tukey’s *post hoc* test for each microbial taxon identified in the LEfSe analysis of adult Wnt1 mice. ∗p < 0.05, ∗∗p < 0.01, ∗∗∗p < 0.001, ns = not significant (p > 0.05).

Supplementary Table 1 Select differentially abundant microbial taxa affected by ethanol (EtOH) treatment in MMTV-Wnt1 and FVB wild-type mice at adolescent and adult stages. Taxa are listed at the genus or species level. Relative abundance changes are indicated for each treatment group. NS: Not significant. E*, E**, E***: Significantly different in post-EtOH-treated samples compared to pre-EtOH treatment, with * indicating significance level (**p* < 0.05, ***p* < 0.01, ****p* < 0.001). ↑: Increased by ethanol treatment. ↓: Decreased by ethanol treatment.
