# Supplementary material for: Effects of alcohol on gut microbiome in adolescent and adult MMTV-Wnt1 mice
Source: Front Oncol. 2025 Jul 16;15:1557040. doi: 10.3389/fonc.2025.1557040 (PMC12307441; doi:10.3389/fonc.2025.1557040)
Supplement: Supplementary Figure 1 — Wilcoxon matched-pairs signed rank test was applied to assess the effects of alcohol exposure on Bacteroides in adolescent Wnt1 mice (left), Anaeroplasma in adolescent Wnt1 mice (right). ∗p < 0.05. [file Presentation1.pptx]

## Slide 1
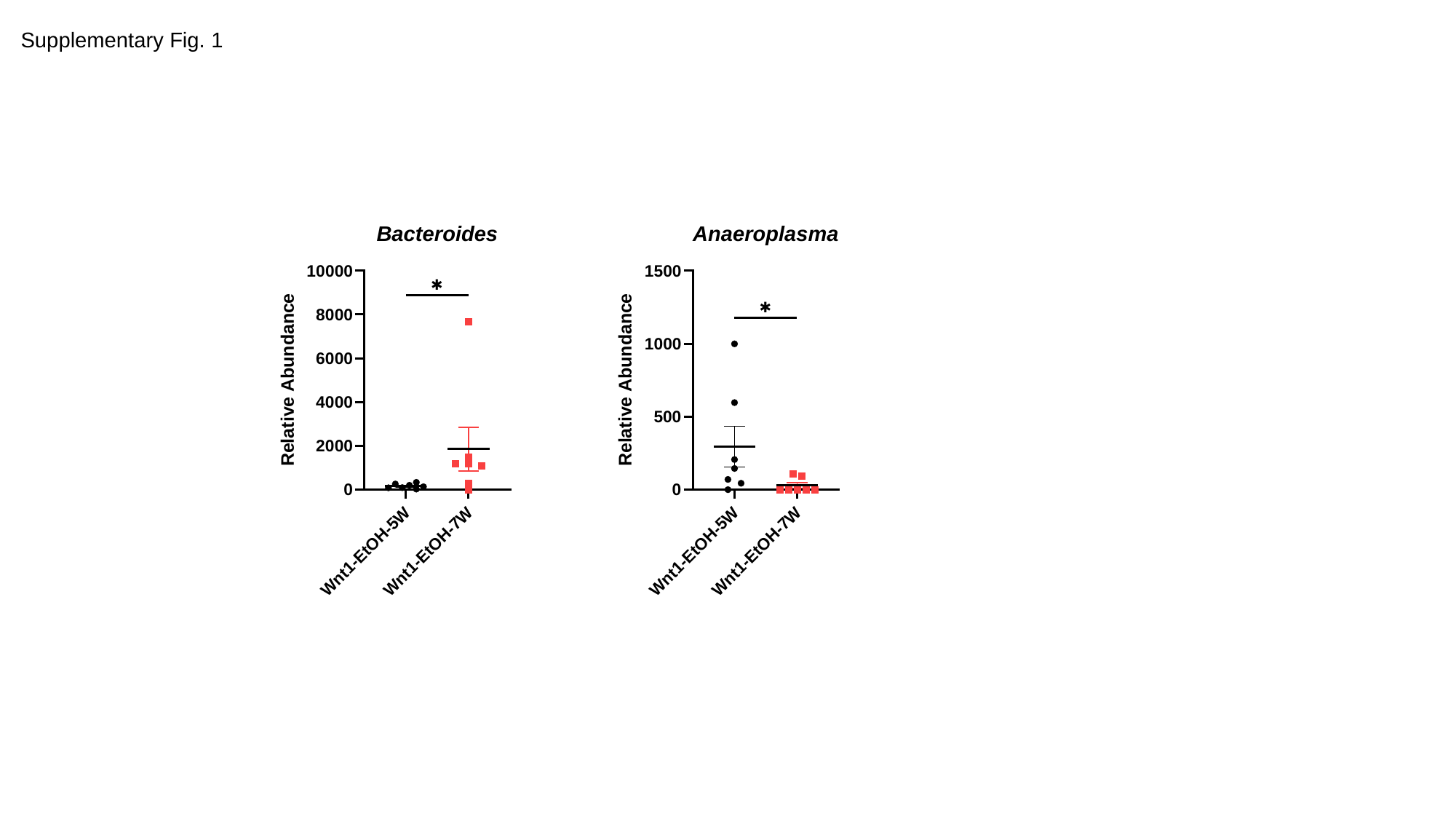

Supplementary Fig. 1

## Slide 2
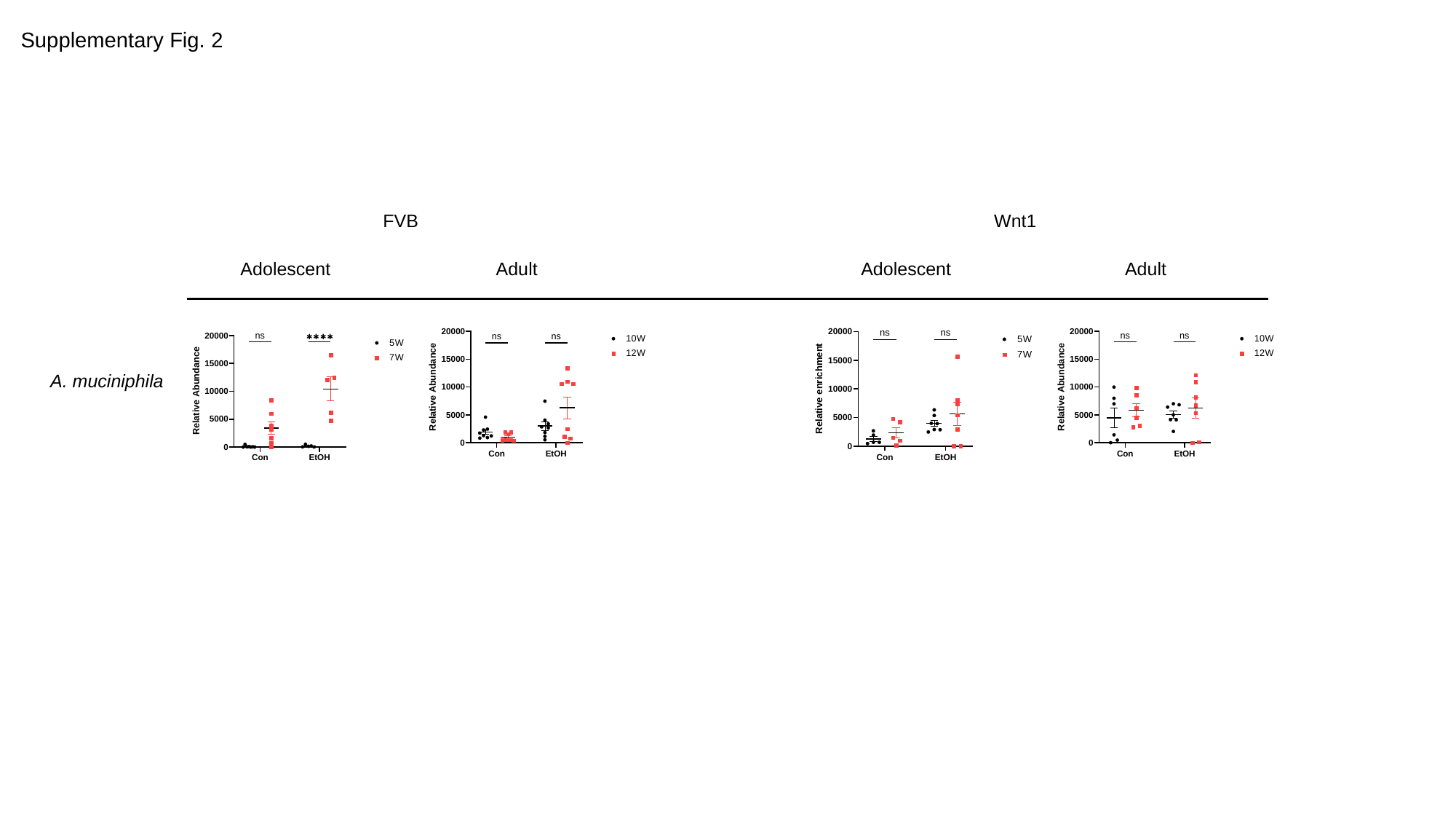

Supplementary Fig. 2
FVB
Wnt1
Adolescent
Adolescent
Adult
Adult
A. muciniphila

## Slide 3
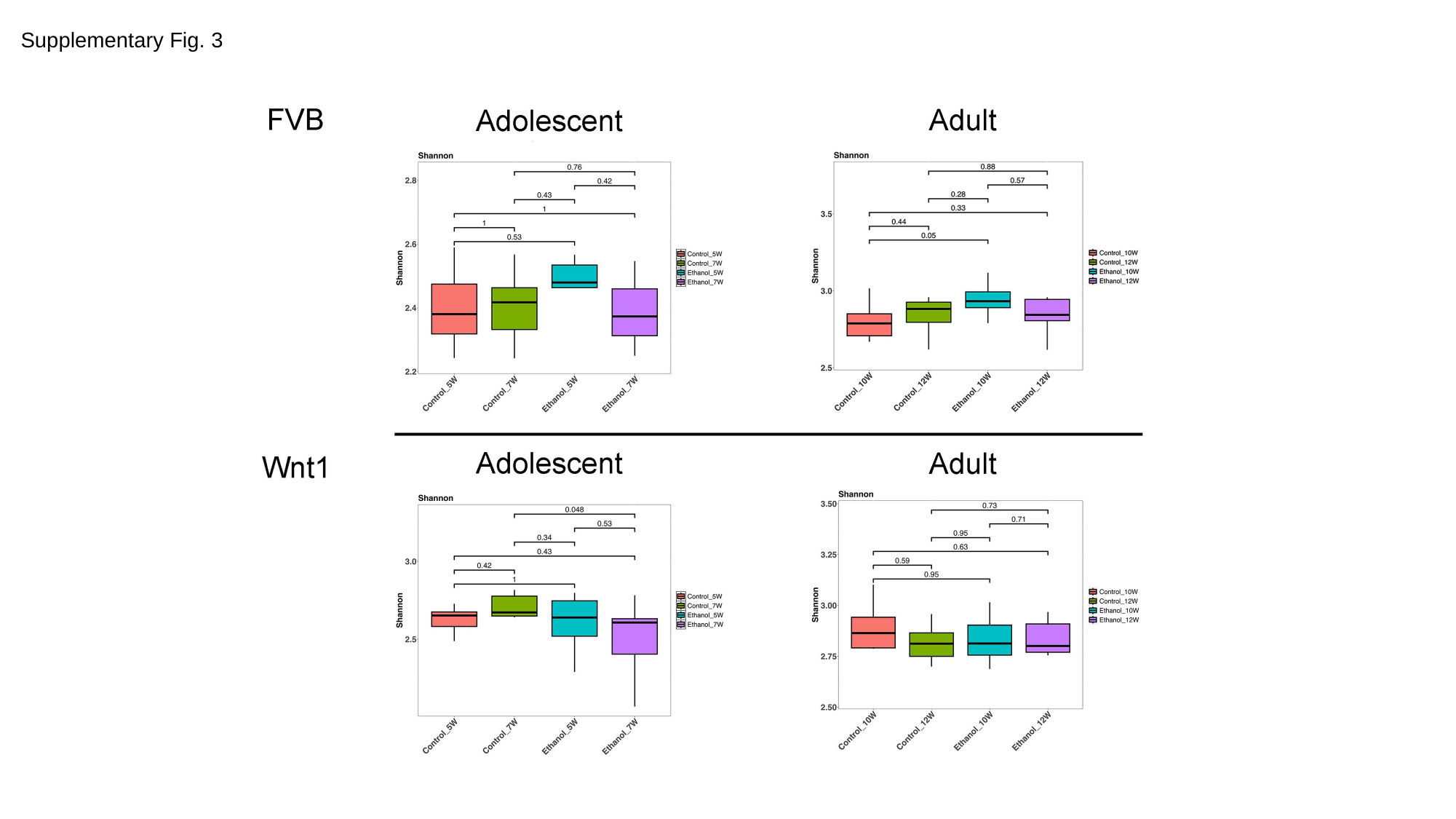

Supplementary Fig. 3

## Slide 4
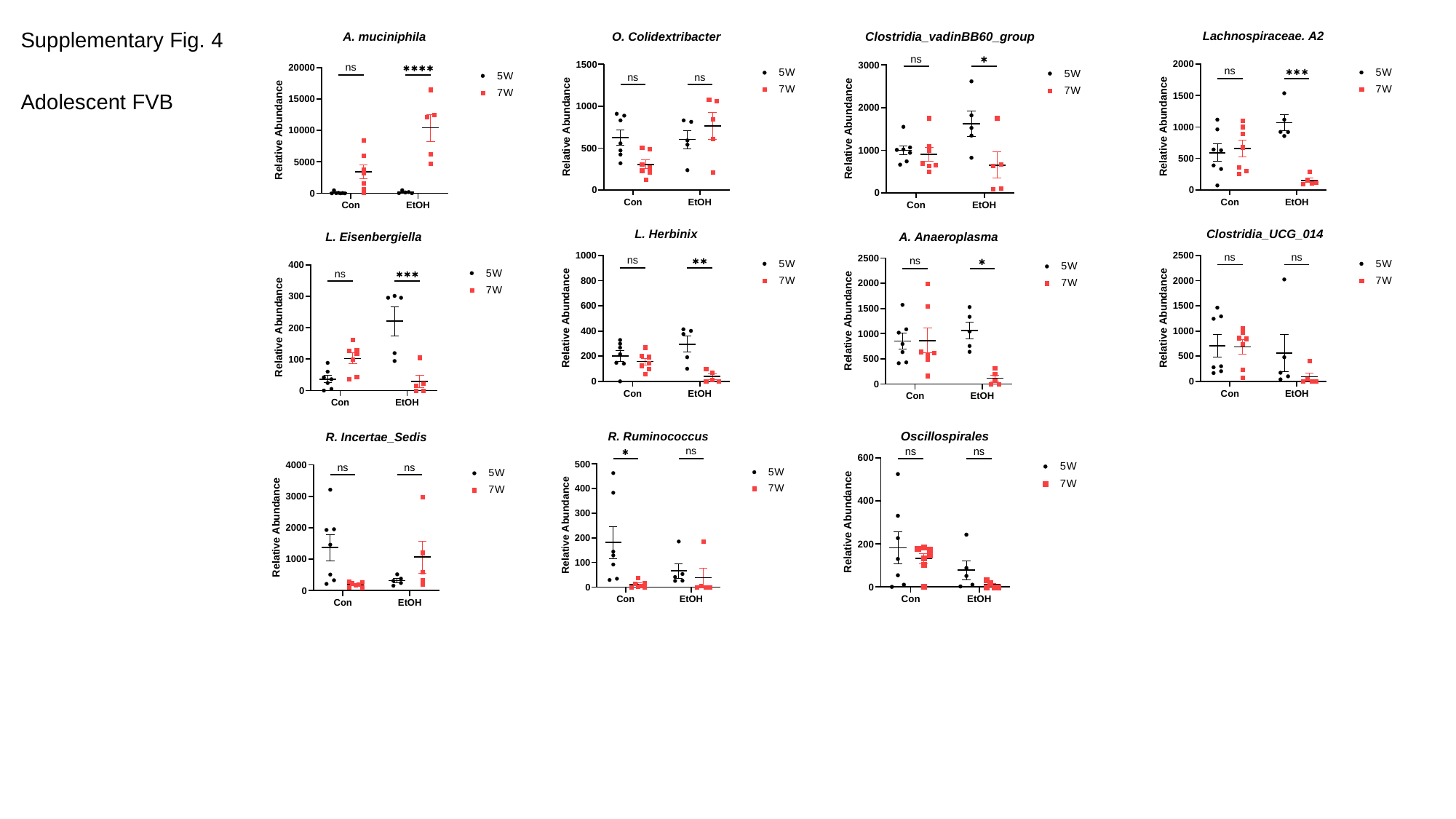

Supplementary Fig. 4
Adolescent FVB

## Slide 5
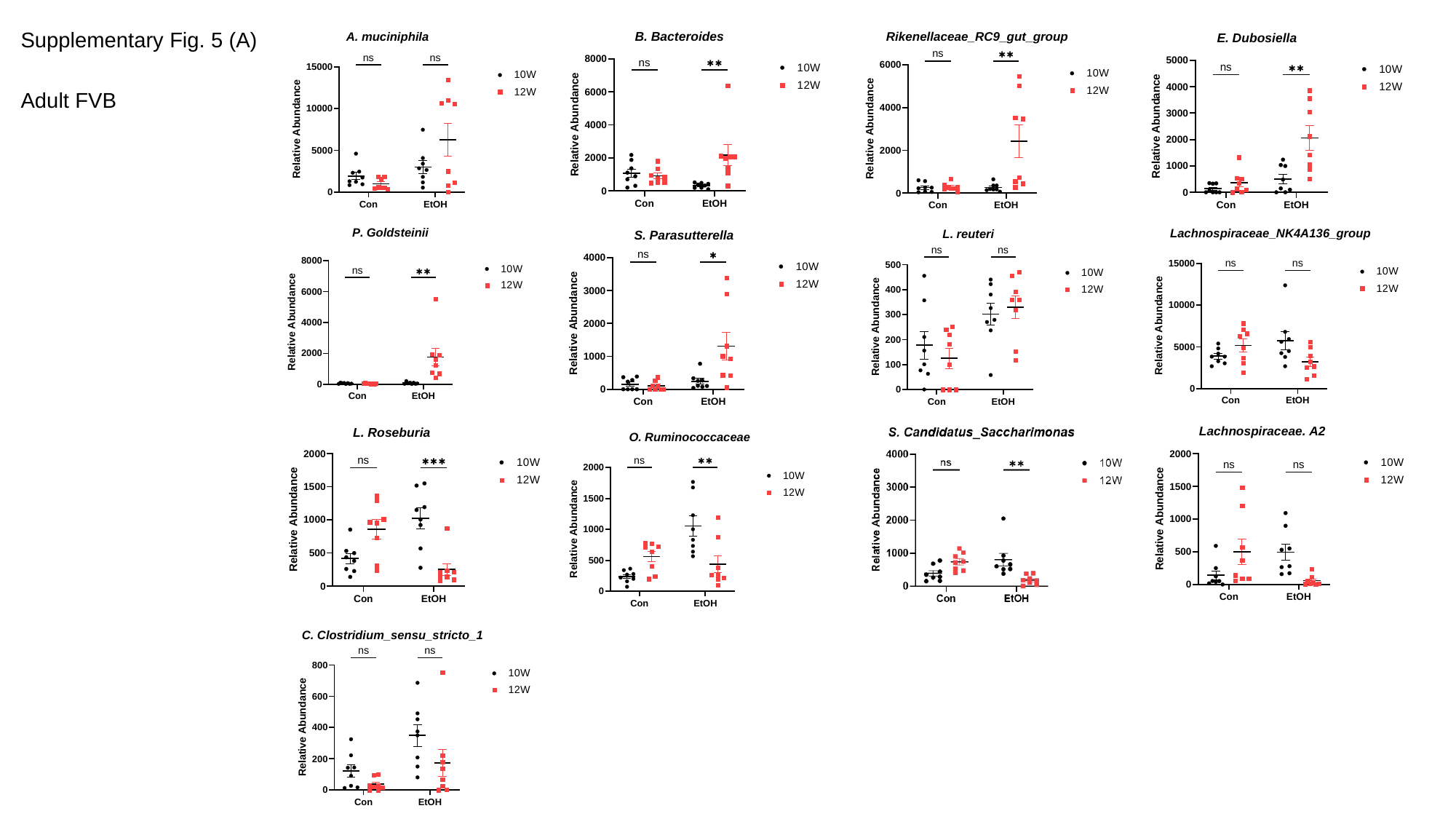

Supplementary Fig. 5 (A)
Adult FVB

## Slide 6
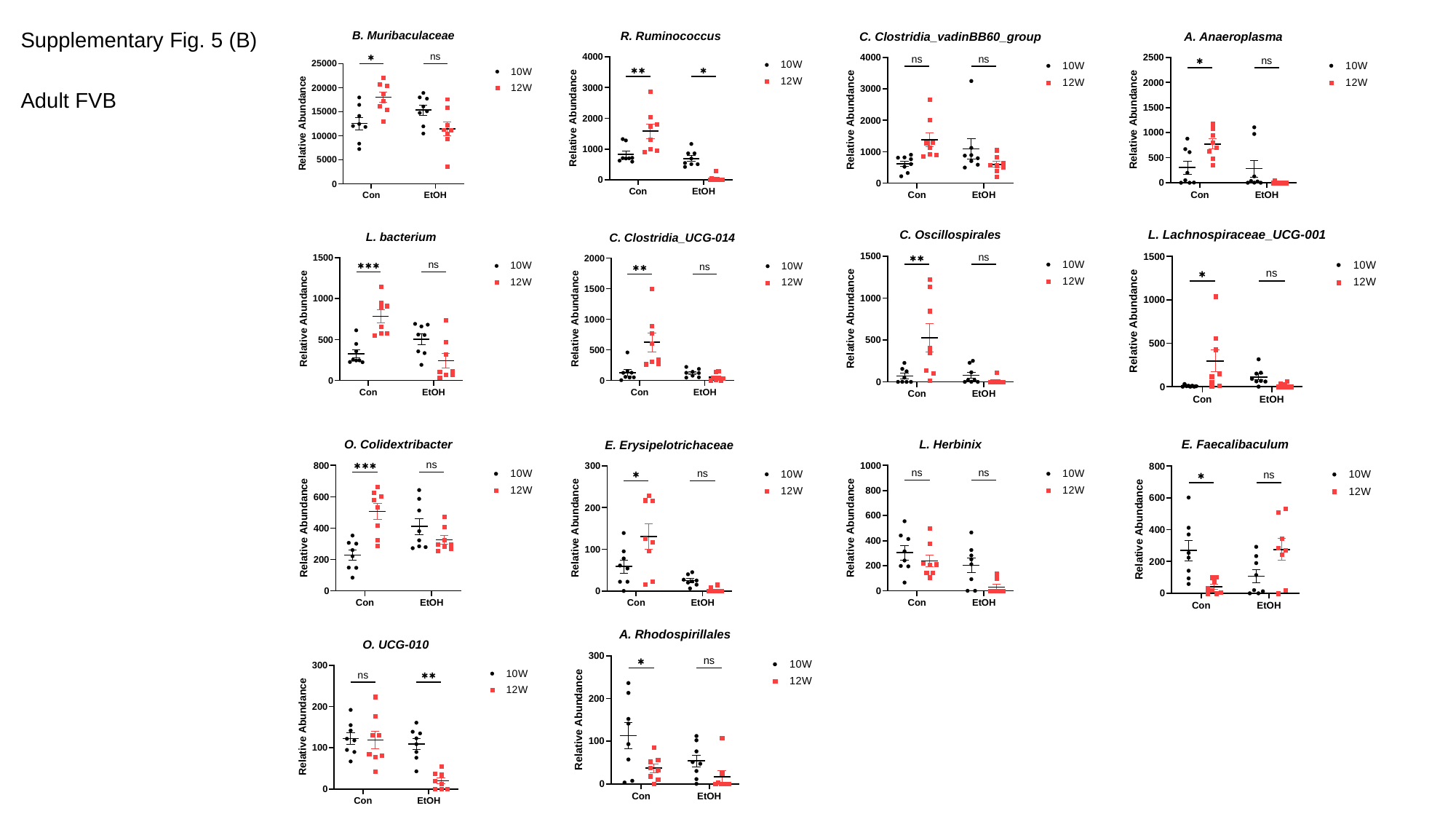

Supplementary Fig. 5 (B)
Adult FVB

## Slide 7
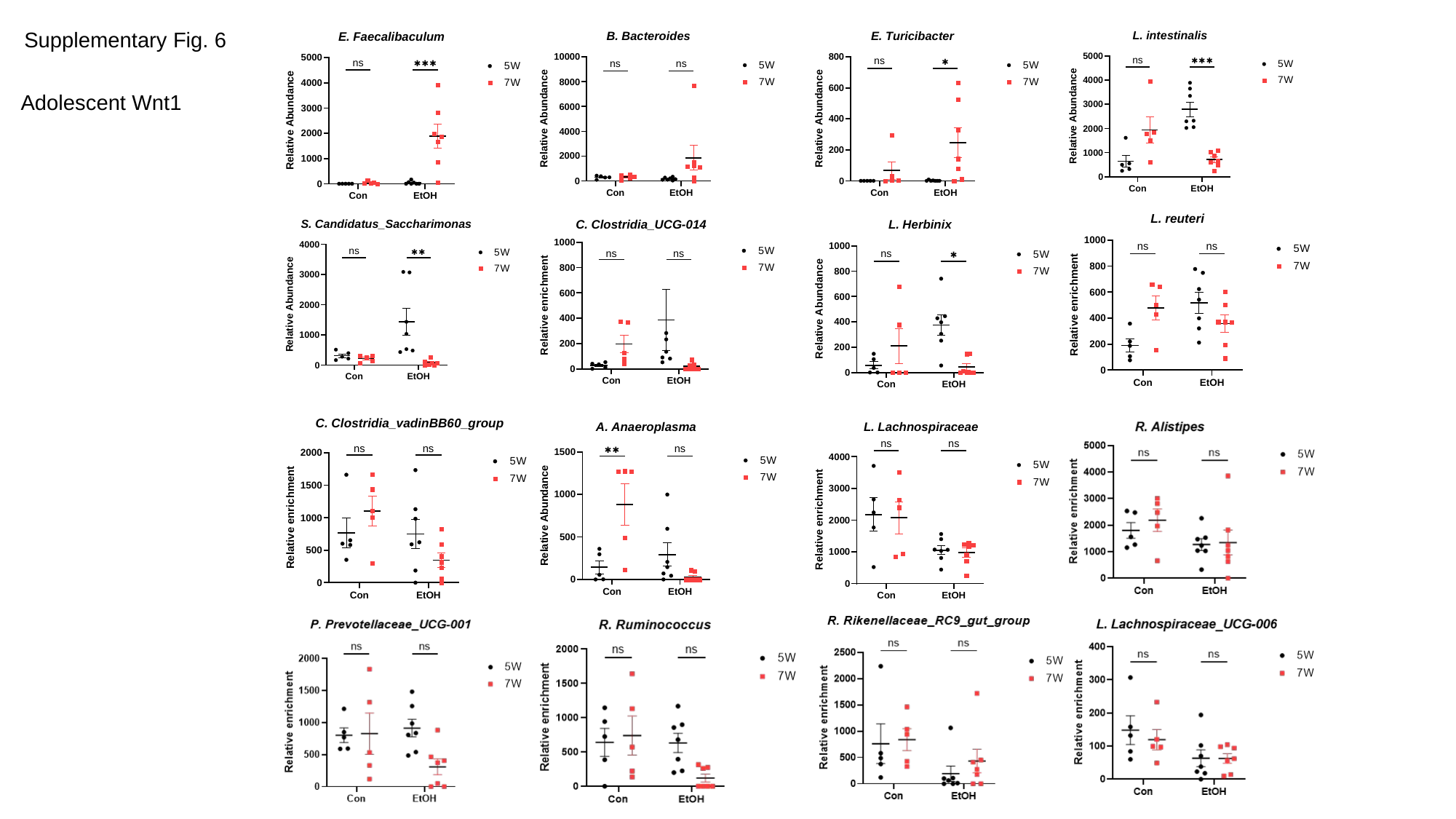

Supplementary Fig. 6
Adolescent Wnt1

## Slide 8
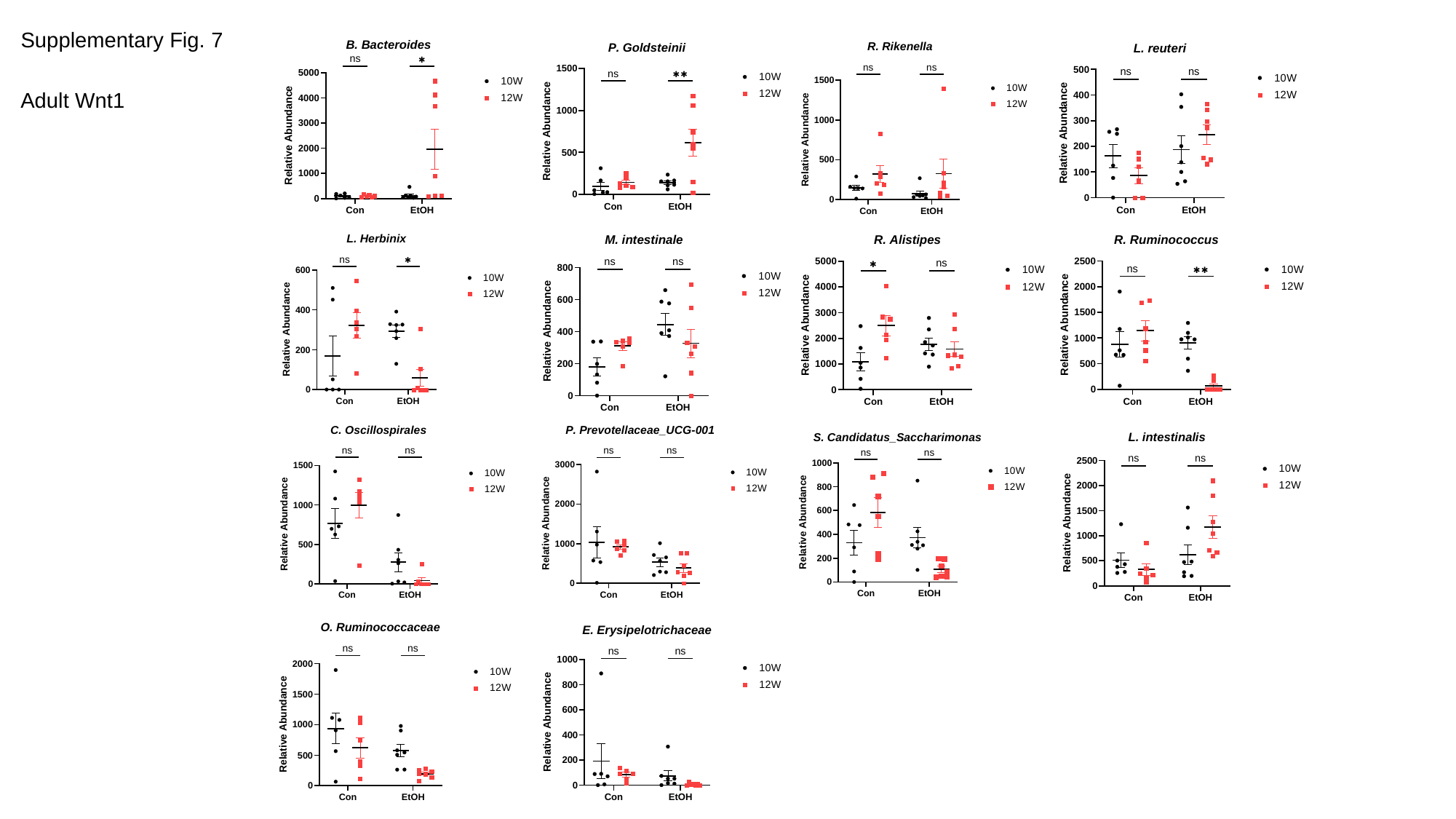

Supplementary Fig. 7
Adult Wnt1

## Slide 9
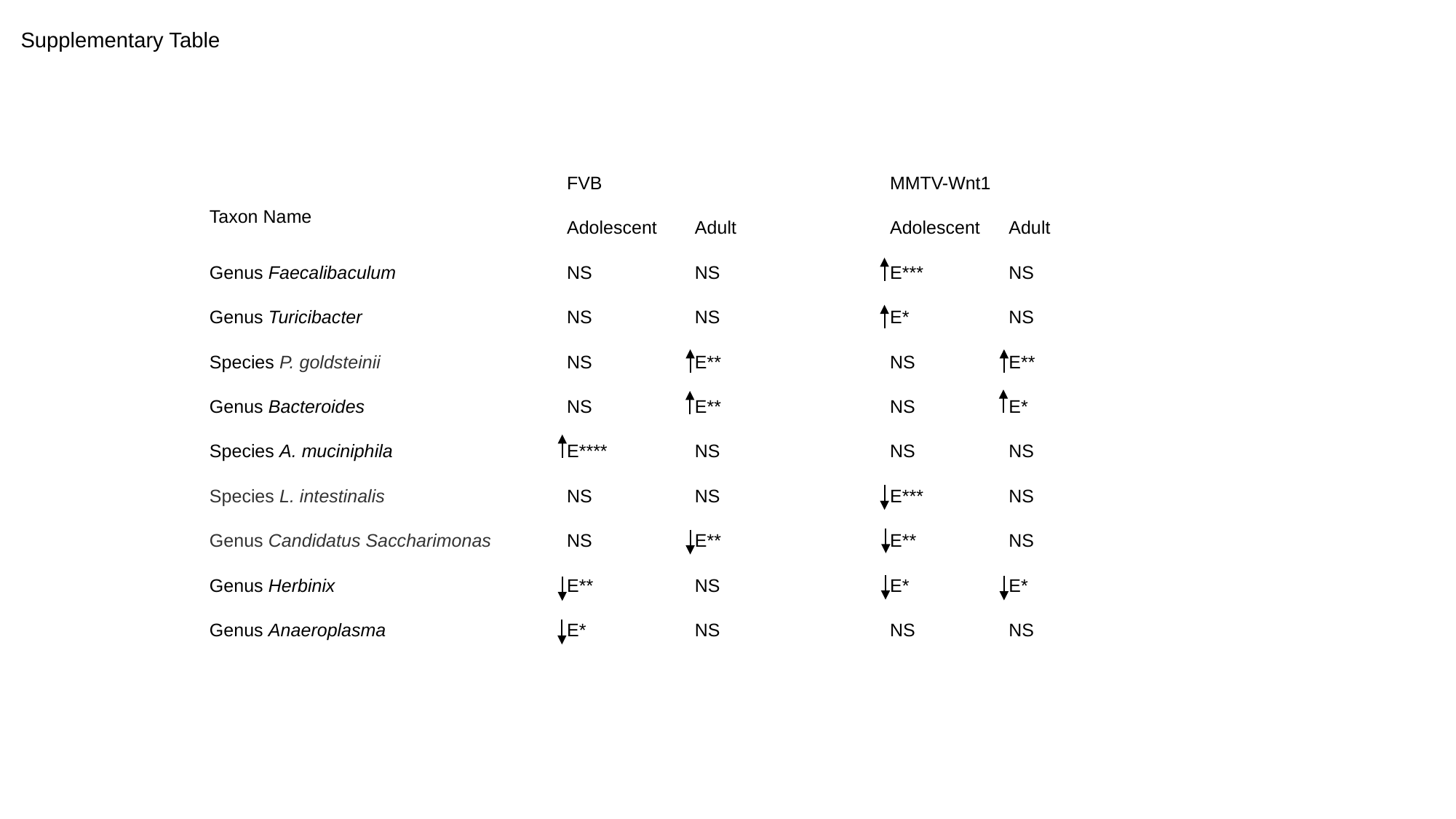

Supplementary Table
| | FVB | | | MMTV-Wnt1 | |
| --- | --- | --- | --- | --- | --- |
| Taxon Name | Adolescent | Adult | | Adolescent | Adult |
| Genus Faecalibaculum | NS | NS | | E\*\*\* | NS |
| Genus Turicibacter | NS | NS | | E\* | NS |
| Species P. goldsteinii | NS | E\*\* | | NS | E\*\* |
| Genus Bacteroides | NS | E\*\* | | NS | E\* |
| Species A. muciniphila | E\*\*\*\* | NS | | NS | NS |
| Species L. intestinalis | NS | NS | | E\*\*\* | NS |
| Genus Candidatus Saccharimonas | NS | E\*\* | | E\*\* | NS |
| Genus Herbinix | E\*\* | NS | | E\* | E\* |
| Genus Anaeroplasma | E\* | NS | | NS | NS |
